# Defining Childhood Severe Falciparum Malaria for Intervention Studies

**DOI:** 10.1371/journal.pmed.0040251

**Published:** 2007-08-21

**Authors:** Philip Bejon, James A Berkley, Tabitha Mwangi, Edna Ogada, Isaiah Mwangi, Kathryn Maitland, Thomas Williams, J. Anthony G Scott, Mike English, Brett S Lowe, Norbert Peshu, Charles R. J. C Newton, Kevin Marsh

**Affiliations:** 1 Kenya Medical Research Institute (KEMRI)–Wellcome Trust Collaborative Research Programme, Centre for Geographic Medicine Research (Coast), Kilifi, Kenya; 2 Centre for Clinical Vaccinology and Tropical Medicine, Churchill Hospital, University of Oxford, Oxford, United Kingdom; 3 Department of Paediatrics, Imperial College, London, United Kingdom; 4 Wellcome Trust Centre for Clinical Tropical Medicine, Imperial College, London, United Kingdom; 5 Department of Paediatrics, The John Radcliffe Hospital University of Oxford, Oxford, United Kingdom; 6 Nairobi KEMRI–Wellcome Trust Collaborative Research Programme, Kenyatta National Hospital, Nairobi, Kenya; 7 Neurosciences Unit, Institute of Child Health, London, United Kingdom; Imperial College, United Kingdom

## Abstract

**Background:**

Clinical trials of interventions designed to prevent severe falciparum malaria in children require a clear endpoint. The internationally accepted definition of severe malaria is sensitive, and appropriate for clinical purposes. However, this definition includes individuals with severe nonmalarial disease and coincident parasitaemia, so may lack specificity in vaccine trials. Although there is no “gold standard” individual test for severe malaria, malaria-attributable fractions (MAFs) can be estimated among groups of children using a logistic model, which we use to test the suitability of various case definitions as trial endpoints.

**Methods and Findings:**

A total of 4,583 blood samples were taken from well children in cross-sectional surveys and from 1,361 children admitted to a Kenyan District hospital with severe disease. Among children under 2 y old with severe disease and over 2,500 parasites per microliter of blood, the MAFs were above 85% in moderate- and low-transmission areas, but only 61% in a high-transmission area. HIV and malnutrition were not associated with reduced MAFs, but gastroenteritis with severe dehydration (defined by reduced skin turgor), lower respiratory tract infection (clinician's final diagnosis), meningitis (on cerebrospinal fluid [CSF] examination), and bacteraemia were associated with reduced MAFs. The overall MAF was 85% (95% confidence interval [CI] 83.8%–86.1%) without excluding these conditions, 89% (95% CI 88.4%–90.2%) after exclusions, and 95% (95% CI 94.0%–95.5%) when a threshold of 2,500 parasites/μl was also applied. Applying a threshold and exclusion criteria reduced sensitivity to 80% (95% CI 77%–83%).

**Conclusions:**

The specificity of a case definition for severe malaria is improved by applying a parasite density threshold and by excluding children with meningitis, lower respiratory tract infection (clinician's diagnosis), bacteraemia, and gastroenteritis with severe dehydration, but not by excluding children with HIV or malnutrition.

## Abstract 2

The accepted definition of severe malaria is appropriate for clinical purposes, but Philip Bejon and colleagues show its specificity in clinical trials may be improved by a parasite density threshold and by excluding children with certain conditions.

## Editors' Summary

**Background.:**

Malaria is responsible for over a million deaths every year, and most of those who die are children in Africa. Until a few years ago, not enough research was being done on malaria, but now many researchers are active in this field. Doctors describe some cases of malaria as being “severe.” Severe malaria in children is very hard to diagnose precisely. Current protocols for diagnosing severe malaria are very sensitive: that is, virtually all children who do have severe malaria will be correctly diagnosed as such. However, the protocols are not very specific: many children who do not have severe malaria, but whose symptoms are instead caused by other diseases, will be defined as suffering from severe malaria. This definition is acceptable for the clinical care of sick children, because it ensures that antimalarial drugs are given to all who might benefit from them, plus some additional children for whom those drugs are not required. However, this definition is not particularly useful for research purposes. When conducting a clinical trial aimed at preventing cases of malaria, it is important to evaluate whether the intervention being tested actually works. Therefore, a more specific method of calculating the number of malaria cases within a population is needed for this type of research.

**Why Was This Study Done?:**

The current definition for diagnosing severe malaria includes a set of signs and symptoms that may be observed at the bedside or as a result of laboratory investigation, along with the detection of malaria parasites in the patient's blood. However, in many malarious areas, a large proportion of the population carries malaria parasites without signs of disease; at the same time, the signs and symptoms of malaria are shared with other diseases. The investigators here wanted to find out whether they could develop an accurate “case definition” of severe malaria that can be used in research.

**What Did the Researchers Do and Find?:**

In this study, two groups of children were studied: first, 1,422 children admitted to the children's wards of the Kilifi District Hospital in Kenya, and second, 4,583 children from the surrounding community. Blood samples were taken in order to find out how common malaria parasites were in the children's blood, and standard clinical and laboratory data were also collected from the children admitted to the hospital. The researchers then compared these data using a computer and tried to find out whether, by excluding certain children who had particular signs, symptoms, or observations, from the diagnosis of severe malaria, they were able to improve the accuracy of their definition. Essentially, for each patient group, the authors calculated “malaria-attributable fractions,” i.e., the proportion of individuals studied whose disease was likely caused by malaria.

The researchers found that in areas with low and moderate transmission of malaria, the proportion of individuals whose disease could be attributed to malaria was high—nearly 85%. In areas with a high transmission rate of malaria, this fraction was much lower, but could be improved by including only children with a high proportion of parasites in their blood. Importantly, the researchers were also able to increase the recognition of children with disease likely caused by malaria by excluding individuals who had also been diagnosed with gastroenteritis, lower respiratory tract infections, meningitis, and bacterial infection in the blood. If all of these individuals were excluded—so only individuals with more than 2,500 parasites per microliter in their blood were regarded as having severe malaria—the “malaria-attributable fraction” rose to 95%.

**What Do These Findings Mean?:**

These findings should not be directly used to change the clinical care of children with the signs and symptoms of severe malaria, but rather can be used within a clinical trial to create a “case definition” of malaria particular to that trial. This ability will help researchers more accurately find out whether the intervention being tested in their trial really does help to prevent cases of malaria or not.

**Additional Information.:**

Please access these Web sites via the online version of this summary at http://dx.doi.org/10.1371/journal.pmed.0040251.

- The World Health Organization Global Malaria Programme details the organization's activities in fighting malaria, and provides a number of helpful resources; a factsheet on children and malaria is also available
- The US Centers for Disease Control and Prevention provides many malaria resources
- General information about malaria, including illustrations, is available from the“Medline Plus” encyclopedia

## Introduction

Severe falciparum malaria is an important end point for preventative intervention trials in its own right, and as a proxy for malaria-associated deaths. The current internationally accepted definition of severe malaria in children is based mainly on the presence of a limited set of bedside or laboratory observations in the presence of parasitaemia with asexual-stage Plasmodium falciparum [1]. This definition was derived from estimates of the associated risks of death [2]. It is biased towards sensitivity, so is appropriate for clinical purposes. However, because the signs on which it is based are common in severe disease of any aetiology, and asymptomatic carriage of parasites is common in endemic areas, the definition lacks specificity. The measurable efficacy and absolute benefit of a preventative intervention depend critically on the specificity of the case definition. Some comorbidities (e.g., malnutrition or HIV) might be risk factors for severe malaria, and other comorbidities (e.g., bacteraemia) might be consequences of severe malaria.

There is no “gold standard” methodology with which to identify individuals with “true” severe malaria rather than severe disease with incidental parasitaemia, so malaria-attributable fractions (MAFs) cannot be calculated by a simple numerator and denominator approach. Instead, we have fitted the risk of severe disease to parasite density as a logistic model, which allows MAFs to be estimated within populations [3]. We have previously used this approach to define a threshold level of parasite density to distinguish asymptomatic parasitaemia (with a coincident, nonmalarial cause of fever) from febrile malaria (that is attributable to the parasitaemia) [4]. Exactly the same principles can be applied to distinguishing severe disease attributable to malaria parasites from severe disease attributable to other causes but having coincident parasitisation. Children admitted to hospital with signs of severe disease [2] were considered “cases,” and asymptomatic children in community studies were considered “controls.” We applied logistic models to determine the effect of a range of comorbidities on MAFs, so as to quantify the effect of excluding these comorbidities on the specificity of the definition of severe malaria. Although MAFs are more widely used to indicate the fraction of febrile cases in the community attributable to malaria [5], here we use MAFs to refer to the fraction of severe disease resulting in hospital admission that is attributable to malaria (and so might be prevented by vaccination).

The case definition that we develop is not intended to limit treatment options for severe malaria, since the balance of risk and benefit will always favour the use of antimalarial drugs and other supportive clinical treatment in a sick child, even when there is a low probability of severe malaria. Rather, it is intended to maximize the discriminatory power of trials conducted to evaluate interventions to prevent malaria. It will also allow more accurate measurement of the disease burden in epidemiological studies.

## Methods

### Location

The KEMRI Centre for Geographic Medicine Research (Coast) is located at Kilifi District Hospital, Kenya. Government-employed clinical officers admit children from the hospital outpatient department to the paediatric ward where research was conducted. The hospital serves ∼240,000 people, who belong mainly to the Mijikenda group and are rural farmers. Children in the surrounding area receive up to 120 mosquito bites infective for Plasmodium falciparum each year [6]. Approval for the inpatient and community based studies was given by the Kenyan Medical Research Institute National Ethics Committee.

### Clinical and Laboratory Methods

From 01 August 1998 to 30 July 2002 we prospectively collected standardised clinical, anthropometric, and laboratory data on all children admitted to the paediatric wards (described in detail elsewhere [7]). Subgroups of severe malaria were examined. Coma was defined as Blantyre coma score below 3. Respiratory distress was defined as the presence of deep breathing. Severe anaemia was defined as a blood haemoglobin level under 50 g/l. MAFs for children with two or more seizures in 24 h and prostration are given separately. These definitions were applied consistently to children at all transmission intensities. Children were weighed on admission using an electronic scale. Weight for age *z* scores were calculated using reference data from the American National Centre for Health Statistics (NCHS) using Epi Info 6.04b (US Centers for Disease Control and Prevention, http://www.cdc.gov/epiinfo/Epi6/EI6dwni.htm). Severe malnutrition was defined as a weight for age *z* score below −3 or bipedal oedema and characteristic skin and hair changes. Lower respiratory tract infection (LRTI) was defined by the clinician's diagnosis on discharge, using a chest X-ray where possible, although WHO integrated management of childhood illness (IMCI) definitions for mild and severe LRTI were also considered [8]. Clinical data were available for all children.

Complete blood counts were performed using an automated counter (Beckman/Coulter, http://www.beckmancoulter.com/). Thick and thin blood smear was stained with 10% Giemsa and examined at 1,000× magnification for asexual forms of P. falciparum and results expressed per microliter using the automated red and white cell indices.

Blood was aerobically cultured for pathogenic bacteria (BACTEC, Becton-Dickinson, http://www.bd.com/) as previously described [7]. Bacterial species likely to represent contamination were excluded from the definition of bacteraemia. Blood cultures were successfully obtained from 100% of the children in this study, with a contamination rate of 14%. Lumbar puncture was guided by a clinical protocol [9], and was conducted on 78% of children with signs of cerebral malaria. Lumbar punctures were not done where there were signs of cerebral herniation or the child died before the lumbar puncture could be performed. Meningitis was defined as a positive CSF culture, a positive CSF latex agglutination test, bacteria seen on CSF Gram stain, or a CSF leukocyte count above 50 cells/μl [10,11]. HIV status was determined at the end of the study using stored, anonymised plasma samples systematically collected for all admissions with an invasive bacterial infection and all admissions after 01 October 1999 using ELISA. PCR was used to confirm infection in children below the age of 18 mo. An HIV status was assigned to 92% of all children with severe malaria admitted after October 1999. Children enrolled before October 1999 were not included in the analysis of HIV comorbidity.

### Clinical Management

Children were managed on the 35-bed general paediatric ward and six-bed high-dependency ward. Children with impaired consciousness or respiratory distress were normally admitted to the high-dependency unit. Children on the high-dependency unit were treated with intravenous quinine and intravenous penicillin and chloramphenicol until the CSF and/or blood culture results were known.

Children with severe anaemia, but without impaired consciousness or respiratory distress, were normally treated on the general paediatric ward. Blood transfusion was given according to WHO guidelines and malaria was treated with sulphamethoxazole-pyrimethamine. Research clinicians provided 24-h clinical cover of both the high-dependency unit and the general paediatric ward, and admissions were reviewed at least daily.

### Community Survey Data

Data were collected between 1997 and 2006 during seven cross-sectional surveys conducted at three different locations within the district. Four were conducting during the rainy season, three during the dry season. The three areas (Ngerenya, Chonyi, and Junju) were chosen to represent relatively low-, moderate-, and high-transmission intensities respectively. Entomological inoculation rates were 10, 22–53, and 42 infective bites/person/year, respectively [6], and the prevalence of asymptomatic parasitaemia was 24.9% (95% CI 23%–27%), 40.5% (95% CI 39%–43%), and 71% (95% CI 66%–76%), respectively [4,12]. The numbers of observations available by age group and location are shown in Table 1. Children who were febrile or unwell at the time of the cross-sectional survey were treated as clinically indicated and excluded from analysis. Data were available for both rainy and dry seasons in each study area. Children in Junju were recruited for a Phase IIb vaccine trial, so limiting the age range and number of children studied [12].

**Table 1:**
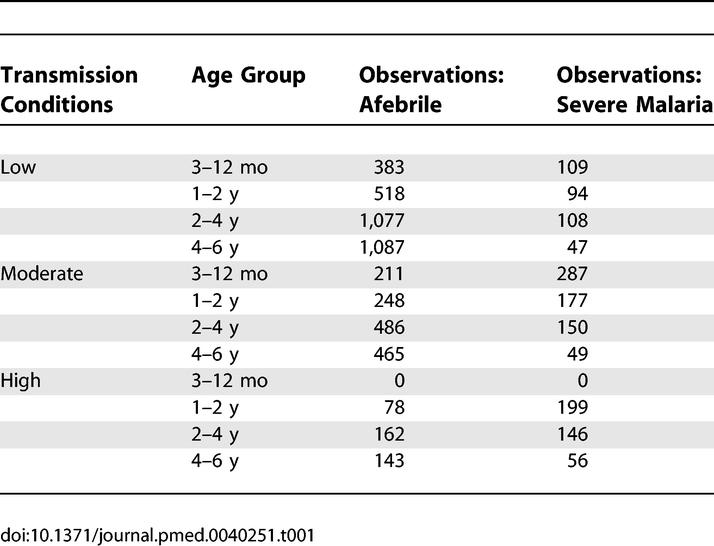
Number of Observations Available for Analysis of Parasitaemia in Afebrile Children from Cross-sectional Surveys and Severe Malaria Cases, by Location and Age Group

### Participants

The initial study was limited to children from three specified locations according to the availability of community cross-sectional surveys. These children were admitted or had blood samples taken in the community between 01 August 1998 and 01 August 2005. 1,422 children were admitted to Kilifi District Hospital with signs of severe disease during this period, and 4,853 samples were taken in the community. The distribution by age and location of these children is given in Table 1.

To study comorbidities and clinical presentations, a more restricted subset of these children was used, for whom detailed clinical information was available. These children were admitted between 01 August 1998 and 30 July 2002. 1,361 children had signs suggestive of severe disease.

### Analysis

Parasite densities from the children with signs of severe disease (considered “cases”) and from children in cross-sectional surveys (considered “controls”) were used to model the relationship between parasite density and severe disease. The method has been described previously [5]. Briefly, a logistic regression model was used in which log(*p*/1 − *p*)= *a* + *bx^τ^*, where *p* is the probability of severe disease (i.e., inpatients with severe disease are treated as “positive” and well children in cross-sectional surveys as “negative”), *x* is the density of parasitaemia, and *τ* is the power function, which improves the numerical stability of the maximum likelihood estimation. The coefficient *b* in the logistic model can then be used to calculate the risk of malaria for each individual. The risks of a group of individuals can be summed to estimate the proportion of the group having “true” severe malaria rather than another cause of severe disease with coincident parasitaemia. This gives the positive predictive value, or MAF, for the group analysed. Sensitivity and specificity can then be calculated from the MAFs.

The model was repeated in different age groups, locations, and for different comorbidity patterns. Confidence intervals (CIs) were estimated by bootstrapping, using 5,000 iterations. The impact of age and location was modelled on a data set covering 1997–2004, restricted to cases of severe disease presenting from the three geographical locations used to establish the cross-sectional datasets. The impact of various comorbidities was studied on all admissions between 1998 and 2002, when systematically collected information on other clinical features was available to define comorbidity. The dataset was not large enough to allow models for comorbidity to be corrected by age, location, and season.

## Results

### Case Definitions by Age and Location

**Figure 1:**
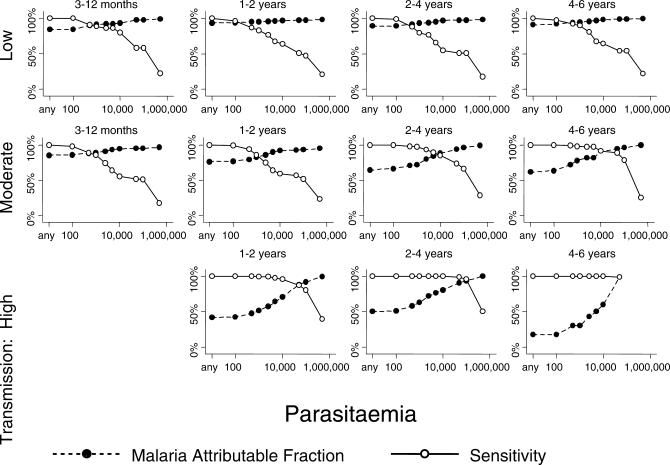
The Attributable Fractions and Sensitivity for Severe Malaria Fractions are shown for varying parasite density thresholds according to age group and location. Malaria-attributable fractions are indicated by closed circles, sensitivity by open circles.

MAFs and sensitivities are shown in Figure 1. At low transmission intensity, MAFs for severe disease with any parasitaemia were 94% (95% CI 90%–98%) for infants and 88% (95% CI 82%–94%) for 2–4 year olds. In the moderate transmission area the MAF for any parasitaemia was 86% (95% CI 77%–95%) in infants, but this fell to 64% (95% CI 51%–77%) in the 2–4 y age group. Under high transmission, MAFs were much lower at 45% (95% CI 30%–60%) in 1- to 2-y-olds. Using a threshold of at least 2,500 parasites/μl to identify cases would have increased the MAFs from 86% to 93% (95% CI 88%–99%) and from 64% to 80% (95% CI 70%–88%) for infants and 2- to 4-y-olds, respectively, in the moderate-transmission area. However, among 1- to 2-y-olds in the high-transmission area, the MAF was only 61% (95% CI 46%–76%) with a threshold of 2,500 parasites/μl, and 89% (95% CI 79%–99%) with a threshold of 50,000 parasites/μl. Although a threshold of 50,000 included nearly 100% of the true malaria cases in the high-transmission area, it reduced sensitivity in the low-transmission areas to only 50%. A threshold of 2,500 parasites/μl would have a sensitivity of 90% or more. Overall, the MAF for all severe malaria cases was 85% (95% CI 84%–86%) at any parasitaemia level, and 93% (95% CI 92%–94%) with a threshold > 2,500 parasites/μl. A threshold of 2,500 parasites/μl gives a sensitivity of 90% or more at moderate- and high-transmission intensities, but 80% in low-transmission areas.

### Case Definitions by Severe Malaria Syndrome

The MAFs (with CIs) for children with different clinical features and comorbidities are shown in Table 2. Among subgroups of severe malaria, the MAFs were highest in coma, intermediate for severe anaemia, and lowest for respiratory distress. The MAFs for children with a history of seizures and for children with impaired consciousness were similar.

**Table 2:**
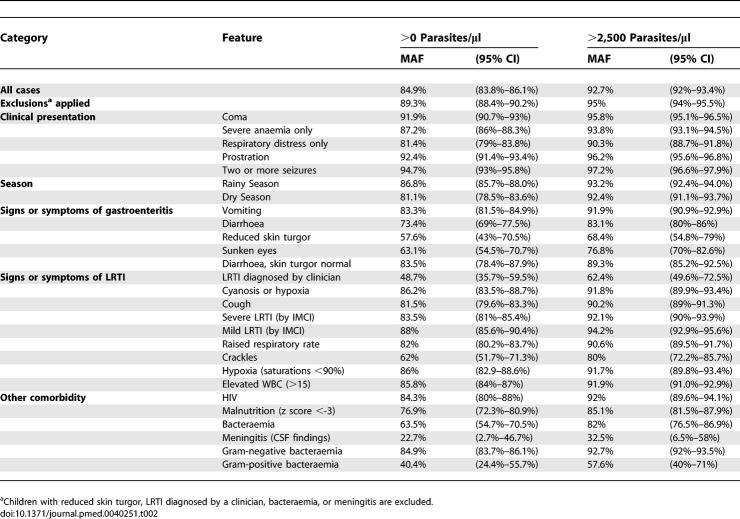
Malaria-Attributable Fractions for Children Admitted with the Clinical Features Given and Any Parasitaemia, or >2,500 Parasites/μl

### Case Definitions by Season

When months associated with greater malaria transmission (“rainy season”) and months associated with lower malaria transmission (“dry season”) were analysed separately, the MAFs for all parasitaemic children were higher during the rainy season, but at a threshold of 2,500 parasites/μl, the MAFs during the rainy and dry seasons were similar.

### Case Definitions by Comorbidity

HIV and malnutrition were associated with only slight reductions in MAFs. The lowest MAFs were seen among children with meningitis, and children with bacteraemia had moderate reductions in MAFs. Gram-negative and gram-positive bacteraemia were associated with markedly different MAFs (Table 2). However, the symptoms and signs of gastroenteritis were associated with differing MAFs. Among children with vomiting, the MAF was similar to that seen for all cases, but a history of diarrhoea (defined as ≥3 loose stools in 24 h) was associated with a lower MAF. However, this reduction was largely accounted for by children with signs of severe dehydration. Among children with documented reduced skin turgor, the MAFs were lower than for children with a history of diarrhoea. Children with diarrhoea but normal skin turgor had MAFs similar to the admissions without comorbidity.

Children for whom a final diagnosis of LRTI was made by a clinician had a low MAF. However, low MAFs were not seen among children who met WHO IMCI criteria for LRTI of any severity. Although a lower MAF was seen among children in whom crepitations were heard on chest auscultation, the MAF among children with a clinician's diagnosis of LRTI was lower still.

The overall MAF rose from 85% (95% CI 83.8%–86.1%) to 89% (95% CI 88.4%–90.2%) after excluding gastroenteritis with severe dehydration, LRTI, meningitis, and bacteraemia, and then to 95% (95% CI 94%–95.5%) when a threshold of 2,500 parasites/μl was used. The sensitivity fell to 80% (95% CI 77%–83%) overall.

## Discussion

Children with falciparum parasitaemia in association with clinical signs of severe malaria had an MAF of 85%, which rose to 95% when a threshold of 2,500 parasites/μl was applied and children with meningitis, lower respiratory tract infection (clinician's diagnosis), bacteraemia, and gastroenteritis with severe dehydration were excluded. Excluding children with HIV or malnutrition did not improve the case definition.

We have previously described severe falciparum malaria in children admitted to hospital by identifying the children at greatest risk of death [2]. However, asymptomatic parasitaemia is common in malaria-endemic areas, and the clinical features of severe malaria can be similar to those of other severe illnesses. A simple clinical definition therefore lacks specificity. This low-specificity definition is appropriate for clinical care, but not for epidemiological studies describing the disease burden or measuring the efficacy of intervention. In previous studies we used the logistic model first proposed by Smith and Schellenberg [5] to define the MAFs among children with uncomplicated fever. The optimal threshold among children aged 1 y and over was 2,500 parasites/μl in the low- and moderate-transmission areas, and any density of parasitaemia in infants [4]. In our analysis of severe inpatient malaria, we found very similar patterns for the variation of thresholds by transmission intensity and age in defining severe malaria.

It is impractical to define a threshold parasitaemia for every age group, village, and season, so any threshold used in a trial or epidemiological study must inevitably be a compromise. Higher thresholds were required in older age groups, and at higher transmission intensities. Data from the high-transmission area support a high threshold (at least 10,000 and perhaps even 50,000 parasites/μl), but this would be associated with a large reduction in sensitivity if applied to the low- and moderate-transmission areas, and transmission may vary considerably even in a restricted area [6]. Based on our overall data set, the best compromise between sensitivity and specificity in defining severe malaria among children below 6 y of age was achieved with a threshold of 2,500 parasites/μl. This threshold reduces sensitivity to 80%, and so may reduce the power of a study. However, a low specificity reduces the apparent efficacy, and so also reduces power. We examined the outcome of these competing effects for our data. If no exclusion criteria or threshold parasitaemia were used on this data set, an intervention with 50% efficacy would have an apparent efficacy of 43% and require *n* = 5,500 for 90% power. After applying exclusion criteria and the threshold of 2,500 parasites/μl, the apparent efficacy would be 48% (*n* = 6,000 for 90% power). Although the apparent efficacy estimate is more accurate (because the vaccine cannot reduce the “nonmalaria” severe disease that is included in the study end point), the sample size has increased slightly because of reduced sensitivity. Were the threshold parasitaemia applied without exclusion criteria, the apparent efficacy would be 46% (*n* = 7,000 for 90% power). However, the effect will depend critically on transmission intensity and the incidence of severe nonmalarial disease (e.g., during a cholera outbreak), so these calculations should be considered illustrative rather than definitive.

Severe malaria comprises three distinct, though often overlapping, clinical syndromes: impaired consciousness, respiratory distress, and severe anaemia [2]. MAFs were highest among children with coma, then severe anaemia, and least with respiratory distress. However, a threshold of 2,500 parasites/μl gave MAFs of over 90% in all syndromes. Children admitted with two or more seizures in 24 h had very high MAFs, suggesting that malaria is the most common cause of convulsions in the children studied.

Children with coexistent HIV or malnutrition should not be excluded from a case definition of severe malaria, as the high MAFs indicate that malaria is often the immediate proximate cause of the life-threatening illness. A history of diarrhoea was associated with reduced MAFs, but this effect was found mainly among children with reduced skin turgor (a sign of intracellular dehydration). Children with diarrhoea but normal skin turgor had a high MAF. Thus, skin turgor appears to distinguish severe dehydration with extravascular fluid depletion in gastroenteritis from intravascular fluid depletion without signs of marked tissue dehydration in malaria [13].

A clinical syndrome of LRTI at admission (according to WHO IMCI guidelines) was not associated with reduced MAFs. However, when clinicians made a final diagnosis of LRTI, they successfully identified a group of children with low MAFs. This is surprising, since the deep breathing produced by malaria-related acidosis is easily confused with respiratory disease [2]. Of the signs and symptoms examined, only crackles on auscultation of the chest were associated with lower MAFs. This observation is also surprising, considering the high interobserver variability for findings on lung auscultation [14,15]. Since clinical criteria alone were insufficient to predict comorbidity, it is likely that the results of investigations contributed significantly to the clinician's diagnoses. However, children with bacteraemia received a primary diagnosis of bacteraemia rather than LRTI, so did not contribute. A high white blood cell count was not associated with a reduced MAF. Data on chest X-ray findings were not available for this analysis, and may have contributed to the overall clinical diagnosis of LRTI. However, this possibility is speculative, and other factors may have contributed to the clinical assessment.

Proven bacterial meningitis should exclude a case from consideration as severe malaria. Among children with bacteraemia the MAFs were reduced, but not by as much as those for meningitis or LRTI. It may be that bacteraemia is the primary cause of severe disease in some children but a consequence of severe malaria in other children. When enteric gram-negative organisms and gram-positive organisms were examined separately, the reduction in MAF was confined to gram-positive organisms. Bacteraemia with enteric organisms may often be a consequence of malaria infection in this group of children, whereas gram-positive bacteraemia may be unrelated.

What are the limitations of this study? Data from all admissions with severe disease during the study period were used to examine the impact of comorbidity, but data used to establish patterns of asymptomatic parasitaemia in the community were necessarily from a subsample, limited geographically and temporally. It is possible that the true distribution of asymptomatic parasitaemia in the whole of Kilifi district might be different from that used in this analysis. Although this might bias the calculated MAFs, this consideration applies equally to each subgroup for which MAFs were estimated. Conclusions based on comparisons between comorbidities and all cases are therefore still justified.

Hospital inpatient studies on the correlation between diseases are vulnerable to Berkson's bias [16]. If, for example, the presence of incidental parasitaemia were to increase the probability of admission among primary cases of malnutrition, then a biased association would occur between malnutrition and parasitaemia. This artefactual association could bias the MAF estimate upward in the malnutrition subgroup. However, for the majority of children admitted to Kilifi District Hospital the malaria slide performed after admission is the first malaria investigation of their illness. If the presence of malnutrition increases the probability of admission among primary cases of malaria, the MAFs would be increased among malnutrition cases. However, the objective of the study was to define a group of patients with a diagnosis of severe malaria in whom the MAF was optimised. Malaria would nevertheless be the immediate proximate cause of admission, and these cases might be prevented by an effective vaccine. As it turned out, some of the more visible conditions with the greatest potential for a biased association (LRTI and gastroenteritis) were also those that had the smallest MAF estimates and were excluded from the final end-point definition.

Data on funduscopy was not available in this study. Retinal changes specific to cerebral malaria are well described [17], but require specialist examination techniques by experienced operators. Further evaluation of direct funduscopy by general clinicians is desirable, but these techniques are unlikely to be part of a case definition in many field sites, and it is difficult to examine the retina fully in conscious children. Furthermore, data were not available in Kilifi during this study. Future studies calculating MAFs should collect data on specialist and nonspecialist funduscopy to evaluate the specificity of the technique in defining severe malaria.

The protocol for HIV testing changed during the study, but the MAF among HIV-positive children was calculated from the group admitted after October 1999. HIV data were available on 92% of these children, and so significant bias seems unlikely.

In an single district in Kenya, with transmission ranging from less than one up to 120 infective bites per year, typical of the majority of transmission setting in sub-Saharan Africa [6,18], a threshold of 2,500 parasites/μl to define severe malaria offers a reasonable compromise between sensitivity and positive predictive value in children below 6 y of age. This approach now needs to be verified in other settings. The threshold parasitaemia is more likely to vary than is the impact of comorbidities, so analysis of children admitted for severe malaria and of community controls in other locations would still be of value even without detailed data on comorbidity. Children with gastroenteritis and severe dehydration (defined by reduced skin turgor), LRTI (defined by chest X-ray and clinical judgement), and proven meningitis should be excluded. Bacteraemia could be included if a higher parasite density threshold is used, but not otherwise. HIV and malnutrition should not be exclusion criteria. In any intervention study, it will be essential to determine the rates of both nonmalaria- and malaria-associated bacteraemia, gastroenteritis, and LRTI, since it is possible that one effect of vaccination will be to convert parasitaemic comorbidity to aparasitaemic comorbidity. An effective vaccination may even alter the appropriate parasitaemia threshold. An analysis of MAFs should be part of the analysis plan for trials or epidemiological studies of severe malaria, conducted separately for the intervention and the control arm of the trial. Where prior data exist, an analysis to confirm that 2,500 parasites per microliter of blood is an appropriate threshold to define severe malaria would be an advantage.

## Abbreviations

CI: confidence interval
CSF: cerebrospinal fluid
IMCI: integrated management of childhood illness
LRTI: lower respiratory tract infection
MAF: malaria-attributable fraction
